# Pyramiding *rpg4*- and *Rpg1*-Mediated Stem Rust Resistance in Barley Requires the *Rrr1* Gene for Both to Function

**DOI:** 10.3389/fpls.2018.01789

**Published:** 2018-12-05

**Authors:** Roshan Sharma Poudel, Abdullah F. Al-Hashel, Thomas Gross, Patrick Gross, Robert Brueggeman

**Affiliations:** Department of Plant Pathology, North Dakota State University, Fargo, ND, United States

**Keywords:** barley, stem rust resistance, genetic mapping, PCR-GBS, iSelect marker

## Abstract

Stem rust, caused by *Puccinia graminis* f. sp. *tritici* (*Pgt*) is an economically important disease of wheat and barley. *Rpg1* is the only resistance gene deployed in Midwestern US barley varieties and provides remarkable resistance to most North American races, except *Pgt* race QCCJB. *Rpg1* is also ineffective against *Pgt* race TTKSK and its lineage that originated in Africa. The barley *rpg4*-mediated resistance locus (RMRL) conferring resistance to *Pgt* races QCCJB and TTKSK was isolated from line Q21861, which is resistant to all known *Pgt* races due to *Rpg1* and RMRL. To develop elite barley varieties RMRL was pyramided into the varieties, Pinnacle and Conlon (both contain *Rpg1*), producing the near isogenic lines (NILs), Pinnacle RMRL-NIL (PRN) and Conlon RMRL-NIL (CRN). The CRN was resistant to *Pgt* races QCCJB (RMRL specific) and HKHJC (*Rpg1* specific) at the seedling stage and *Pgt* race TTKSK (RMRL specific) at the adult stage. In contrast, PRN was susceptible to QCCJB and HKHJC at the seedling stage and TTKSK at the adult stage. Interestingly, PRN’s susceptibility to QCCJB and HKHJC showed that RMRL was non-functional in the Pinnacle background but its presence also suppressed *Rpg1*-mediated resistance. Thus, in the absence of a gene/s found in the Q21861 background, *Rpg1* becomes non-functional if RMRL is present, suggesting that another polymorphic gene, that we designated *Rrr1* (required for *rpg4*-mediated resistance 1), is required for RMRL resistance and *Rpg1*-mediated resistance in the presence of RMRL. Utilizing a PRN/Q21861 derived recombinant inbred line (RIL) population, *Rrr1* was delimited to a ∼0.5 MB physical region, slightly proximal (∼1.8 MB) of RMRL on barley chromosome 5H. A second gene, designated required for *Rpg1*-mediated resistance 2 (*Rrr2*), with duplicate gene action to *Rrr1* in *Rpg1*-mediated resistance function, was genetically delimited to a physical region of ∼0.7 MB, slightly distal (∼3.1 MB) to *Rpg1* on the short arm of barley chromosome 7H. Thus, *Rrr1* is required for RMRL resistance and *Rrr1* or *Rrr2* is required for functional *Rpg1*-mediated resistance in the presence of the RMRL introgression. Candidate *Rrr1* and *Rrr2* genes were identified that need to be considered when pyramiding *Rpg1* and RMRL in barley.

## Introduction

The obligate biotrophic fungus *Puccinia graminis* f. sp. *tritici* Eriks. and E. Henn. (*Pgt*) is the causal agent of stem rust (or black rust) in bread wheat (*Triticum aestivum* L.) and barley (*Hordeum vulgare* L.). Historically, stem rust epidemics caused devastating yield losses of wheat and barley in several parts of the world, including the northern Great Plains of the United States (Roelfs, 1992; Steffenson, 1992). The northern Great Plains is a stem rust prone area that regularly suffered stem rust epidemics in barley prior to the 1940s (Steffenson, 1992). In 1942, the barley variety Kindred was released as the first commercial barley containing the stem rust resistance gene *Rpg1* (Resistance to *P. graminis 1*) (Steffenson, 1992) and since then, barley-breeding programs in the upper Midwest of the United States have fixed the *Rpg1* gene in their lines, which originally came from either cv. Peatland (CIho 5267), Chevron (Ciho 111) or Kindred (Ciho 6969) (Steffenson, 1992; Jin et al., 1994a; Zhou et al., 2014). Thus, for nearly 80 years, *Rpg1* has been the only source of durable resistance in barley and has maintained its effectiveness against the majority of isolates making up the North American *Pgt* populations.

In 1989, a new race of *Pgt*, designated as QCC [later designated as QCCJB (Jin et al., 2008)] was identified in North Dakota (ND) that was virulent on barley cultivars carrying *Rpg1* (Roelfs et al., 1991). The *Pgt* race QCCJB became the most prevalent race in the northern US, threatening regional commercial barley varieties with potential stem rust epidemics that had not been experienced for nearly 50 years (Roelfs et al., 1993). To tackle this threat, Jin et al. (1994a) screened 18,000 barley accessions from the USDA National Small Grains collection (Aberdeen, ID) and identified the unimproved barley line Q21861 as a remarkable source of resistance against QCCJB. Genetic studies utilizing several biparental populations derived from Q21861 and different susceptible barley lines characterized a temperature sensitive and recessive gene designated *rpg4* in the line Q21861 conferring resistance to *Pgt* race QCCJB (Jin et al., 1994b). The *rpg4* gene was mapped to the sub-telomeric region of the long arm of barley chromosome 5H (Borovkova et al., 1995). Q21861 also contains a dominant resistance gene that confers resistance to the rye stem rust pathogen *P. graminis* f. sp. *secalis* (*Pgs*) isolate 92-MN-90 that was originally reported to co-segregate with *rpg4*, and was designated as *Rpg5* (Sun et al., 1996; Brueggeman et al., 2008). A map-based cloning approach identified the dominant rye stem rust resistance gene *Rpg5* as a nucleotide-binding site leucine-rich repeat-serine/threonine protein kinase (NBS-LRR-STPK) domain protein (Brueggeman et al., 2008). The original high-resolution map determined that *Rpg5* was independent of, but tightly linked to *rpg4* and colocalized to a 70 kb physical region (Brueggeman et al., 2008). Utilizing the high-resolution mapping populations and additional single nucleotide polymorphism (SNP) markers, the *rpg4*/*Rpg5* locus was refined. In two of the high-resolution populations the genes colocalized to the 70 kb region containing the *Rpg5* rye stem rust resistance gene and was delimited to three tightly linked genes within the region; the NBS-LRR gene *HvRga1*, the NBS-LRR-STPK gene *Rpg5*, and an actin depolymerizing factor-like (Adf) gene, *HvAdf3* (Wang et al., 2013). Post-transcriptional gene silencing utilizing barley stripe mosaic virus (BSMV) virus-induced gene silencing (VIGS) of each of the genes delimited to the *rpg4*/*Rpg5* locus determined that all three genes, *HvRga1*, *Rpg5* and *HvAdf3* are required together to confer *rpg4*-mediated stem rust resistance. Thus, this complex of genes required for wheat stem rust resistance was designated the *rpg4*-mediated resistance locus (RMRL). However, a study of multiple alleles of *Rpg5*, *HvRga1* and *HvAdf3* determined that *Rpg5* is the only gene that contains polymorphisms that explain resistance—vs—susceptibility, therefore, *Rpg5* appears to be the resistance gene with functional polymorphism at the locus, yet *HvRga1* and *HvAdf3* are required for resistance (Arora et al., 2013). Interestingly, the major allele found in cultivated and wild barley lines collected from around the world (Arora et al., 2013; Steffenson et al., 2017) contain a PP2C domain in place of the Rpg5-STPK domain in most *Pgt* race QCCJB susceptible barley lines that result in the loss of RMRL-mediated resistance. Thus, markers based on this polymorphism are used to track RMRL introgression via marker-assisted selection (Derevnina et al., 2014).

Currently, the highly virulent *Pgt* race TTKSK (also known as Ug99) and its lineage pose an alarming threat to global wheat and barley production and world food security (Steffenson and Jin, 2006; Singh et al., 2011; Steffenson et al., 2017). Race TTKSK was first reported in 1999 from wheat fields in Uganda, Africa (Pretorius et al., 2000). The extensive monitoring of this race showed a rapid spread of TTKSK and its evolving lineage throughout other countries in Africa and jumped the red sea into Asia (Singh et al., 2011). The varieties grown on approximately 80–95% of wheat acreage world-wide and the majority of breeding materials were found to be moderately to highly susceptible to TTKSK and its lineage (Singh et al., 2006, 2011). Recently, Steffenson et al. (2017) reported 96% of 2,913 barley accessions, including those containing *Rpg1*, were extremely vulnerable to this evolving *Pgt* race. The reliance on a single resistance gene in the Upper Midwestern US and identification of North American race QCCJB, and the threat of the emergence of African race TTKSK and its lineage leaves barley production in the major barley production region of US and the world vulnerable to potential stem rust epidemics. In barley, RMRL is the only well characterized locus that provides resistance to QCCJB, TTKSK and its lineage (Steffenson et al., 2009), thus, introgression of RMRL into commercially grown barley varieties is important to minimize the risk of stem rust races that have overcome the remarkably durable *Rpg1*-mediated resistance.

The primary goal of barley-breeding programs is to release varieties that are high yielding, have good quality, are stable across dynamic environments and contain resistance to important pathogens or pests (Horsley et al., 2009). Conlon and Pinnacle are recommended malting barley varieties grown in the Upper Midwestern United States (United States Department of Agriculture [USDA], 2012). Like all Midwestern barley varieties, Conlon and Pinnacle are known to contain only the *Rpg1*-mediated stem rust resistance gene, thus, are prone to potential stem rust epidemics from *Pgt* races like QCCJB or TTKSK if or when it was to arrive in the US. Thus, a marker assisted backcrossing scheme was initiated to introgress RMRL into these lines. The Conlon RMRL-near isogenic line (NIL) and Pinnacle RMRL-NIL (PRN) carrying both *Rpg1* and the RMRL were developed via marker-assisted selection. Field trials in Kenya using *Pgt* race TTKSK showed that only the Conlon RMRL-NIL (CRN) gained resistant to *Pgt* race TTKSK at the adult plant stage in the field but the PRN did not. Corresponding with the adult plant reactions in the field, the CRN exhibited resistance to *Pgt* race QCCJB while the PRN was susceptible to *Pgt* race QCCJB in growth chamber seedling resistance assays. Intriguingly, a later growth chamber assay of the PRN using the *Rpg1* avirulent *Pgt* race HKHJC showed that the PRN not only lacked RMRL-mediated resistance but had also lost *Rpg1*-mediated stem rust resistance, even though the *Rpg1* gene was fixed.

Arora et al. (2013) sequenced *Rpg5* alleles from a diverse set of barley accession and categorized them into four resistant and four susceptible groups based on polymorphisms present in the functional *Rpg5* and non-functional *rpg5* alleles. Pinnacle and Conlon, carry the non-functional *rpg5* allele that contains a C-terminal protein phosphatase 2C (PP2C) integrated domain in place of the C-terminal protein kinase domain present in the functional Rog5 alleles. Thus, these alleles belong to either the group 2 or group 3 susceptible genotypes as described by Arora et al. (2013). A combination of Rpg5-LRR/STPK and Rpg5-LRR/PP2C specific sequence tag site polymerase chain reaction (PCR) markers were used for marker assisted selection to confirm the presence of full-length Q21861 like *Rpg5-STPK* alleles in elite barley backgrounds from breeding programs around the world, including our introgression into the Conlon and Pinnacle backgrounds. The single *Rpg5* marker was considered sufficient to track the entire RMRL containing *HvRga1*, *Rpg5* and *HvAdf3* as genotyping of over 5,000 recombinant gametes did not identify a single recombinant separating the three genes (Brueggeman et al., 2008; Wang et al., 2013) and the *HvRga1* and *HvAdf3* genes are highly conserved across resistant and susceptible genotypes (Arora et al., 2013). Also, the *Rpg1* gene in all resistance sources in Midwestern breeding programs including the NDSU breeding program that produced Conlon and Pinnacle are identical to the *Rpg1* gene found in Q21861, thus, *Rpg1* is fixed in all lines utilized in the pyramiding scheme. However, despite the presence of the Q21861 like RMRL, and intact *Rpg1* in the PRN, the loss of resistance to both *Rpg1* and RMRL avirulent *Pgt* races HKHJC and QCCJB, respectively, at the seedling stage and TTKSK at the adult stage in the field should be due to a gene/s that are required for resistance but are non-functional in the line Pinnacle. These data suggest the existence of other gene/s required for *Rpg1* and/or RMRL stem rust resistance with natural polymorphism in the primary barley germplasm pool which must be considered when pyramiding these two effective stem rust resistance genes that when present together confer resistance to all known stem rust races.

The aim of this study was to identify the region of the barley genome that segregates for genes required for effective stem rust resistance when RMRL and *Rpg1* are combined in different barley backgrounds. To this end a F_4:5_ recombinant inbred line (RIL) mapping population was developed from the cross between the PRN/Q21861 using single-seed descent to map the other genes required for resistance since both RMRL and Rpg1 are fixed in the population thus the only genes to segregate would be the other genes required for resistance. Utilizing this biparental mapping population, two genes, *Rrr1* (required for *rpg4*-mediated resistance 1) and *Rrr2* (required for *Rpg1*-mediated resistance 2) were mapped to barley chromosomes 5H and 7H, respectively. The genetic and phenotypic analysis revealed that a functional *Rrr1* is required for RMRL resistance yet neither gene is required for *Rpg1*-mediated resistance in the absence of the RMRL. However, when RMRL is added to the genetic background then *Rrr1* or *Rrr2* are required for the *Rpg1*-mediated resistance to function. Thus, based on results from this study, we hypothesize that the introgression of RMRL requires *Rrr1* and to pyramid RMRL with *Rpg1* a functional *Rrr1* or *Rrr2* is necessary to maintain the *Rpg1*-mediated resistance function.

## Materials and Methods

### Development of Pinnacle and Conlon RMRL-NIL

The Pinnacle and CRNs were originally developed to provide elite regional (Upper Midwestern US) adapted germplasm that contained broad resistance to all known stem rust races. The Pinnacle and CRNs were developed via a backcrossing and marker assisted selection scheme. Pinnacle, a two-row malting cultivar developed by the NDSU barley-breeding program and released by the North Dakota Agricultural Experiment Station in 2006, was crossed with the unimproved two-row barley line Q21861 as the RMRL donor. Conlon is also a two-row malting barley variety developed by the NDSU barley-breeding program that was an earlier release by the North Dakota Agricultural Experiment Station in 1996. Conlon was crossed with the Harrington x Q21861 BC_3_F_2_ line designated HQ1 as the RMRL donor. The HQ1 line was developed by backcrossing to the recurrent parent Harrington to the BC_3_ generation using *rpg5-PP2C* allele specific STS markers (primers R5iN-F2 and PP2C-R3) for MAS to insure introgression of RMRL into a Northwest US and Canadian adapted malting barley variety (Brueggeman et al., 2008). The susceptible variety Harrington used to develop the HQ1 line is a two-row malting variety that contains no known stem rust resistance genes and was developed at the University of Saskatchewan (Harvey and Rossnagel, 1984).

The Pinnacle and Conlon RMRL introgression was accomplished by backcrossing BC_1_F_1_ derived individuals at each generation to the recurrent elite malting background that were subsequently selfed to generate F_2_ progeny. Approximately 12–15 F_2_ individuals from each cross were genotyped using the RMRL specific markers (described below in *Marker assisted selection for RMRL*) and progeny homozygous for RMRL were selected. The homozygous *rpg5*-STPK/RMRL selected F_2_ individuals from the Pinnacle and Conlon backcrosses were again crossed to the recurrent parents. The backcrossing, selfing and marker assisted selection for RMRL was repeated twice for Pinnacle and thrice for Conlon to develop, BC_3_F_1_ Pinnacle and BC_4_F_1_ Conlon, individuals, respectively. The final backcrossed individual was selfed to identify BC_3_F_3_ Pinnacle (Q21861/4^∗^Pinnacle F_3_) and BC_4_F_3_ Conlon (Q21861/4^∗^Conlon F_3_) NILs containing both *Rpg1* and RMRL in the homozygous state.

### Marker Assisted Selection for RMRL

Two previously designed dominant markers, Rpg5-LRK and Rpg5-LRK/PP2C were used to detect the presence of functional *Rpg5*-*STPK* allele and absence of the *rpg5-PP2C* allele, respectively (Derevnina et al., 2014). Genomic DNA (gDNA) was extracted from leaf samples using the protocol described in Richards et al. (2016). PCRs were performed using a Mastercycler Pro programmable thermocycler (Eppendorf, Hauppauge, NY, United States) programmed for 95°C for 4 min; followed by 35 cycles of 95°C for 30 s, 62°C for 1 min, and 72°C for 1 min; followed by 72°C for 5 min. PCR amplifications were performed in a 20 μl reaction volume containing 10 ng of template gDNA, 1× GoTaq^®^ Flexi Buffer (Promega, United States), 1 mM MgCl_2_, 0.15 mM dNTP mix, 0.4 μM of each forward and reverse primer, 1.25 μ GoTaq^®^ DNA polymerase. BC_n_F_2_ lines were selected for further backcrossing if they amplified a 1,046-bp fragment from *Rpg5-STPK* alleles utilizing Rpg5-LRK markers but had no amplification for the *rpg5-PP2C* allele as indicated by an 840 bp amplicon when utilizing the Rpg5-LRK/PP2C marker.

### Development of RIL Mapping Population

To develop the PRN/Q21861 RIL mapping population with fixed RMRL and *Rpg1* genes to map the gene/s involved in RMRL- and *Rpg1*-mediated resistance pathway, the PRN (RMRL+/Rpg1+) was crossed with barley accession Q21861 (RMRL+/Rpg1+). The F_1_ generated from the PRN/Q21861 cross was self-fertilized and F_2_ individuals were advanced to the F_4:5_ generation by single-seed descent (Brim, 1966) to generate a 126 individual RIL population. At the F_2_ stage, 94 of the 126 individuals that were advanced to develop the RIL were phenotyped using *Pgt* race QCCJB to determine the mode of inheritance of *Rrr1* (rust inoculation explained in rust assay section). These individuals were assayed at 14 days post inoculations and later transplanted from cones to 6-inch pots to grow along with other remaining F_2_ individuals to self-fertilize for RIL population advancement.

### Stem Rust Disease Phenotypic Assays

#### Field Assay

To identify NILs exhibiting TTKSK resistance, four F_2_ derived PRN individuals and 12 F_2_ derived CRN individuals were evaluated in the International Stem Rust Nursery at the Kenyan Agricultural Research Institute in Njoro, Kenya during the 2015 field season (Supplementary Table S1). The parental lines (Q21861, Pinnacle and Conlon), susceptible control (Steptoe) and the spreader rows for rust inoculum were planted as described in Zurn et al. (2014). The entries were planted in two replications and the screening was done at the adult plant stage. The modified Cobb scale was used to record the disease severity of stem rust (Peterson et al., 1948; McIntosh et al., 1995). Based on the size and type of the uredinia, the infection response (IR) were categorized into resistant (R; small uredinia surrounded by chlorosis or necrosis), moderately resistant (MR; medium sized uredinia surrounded by chlorosis or necrosis), moderately susceptible (MS; medium to large size uredinia without chlorosis or necrosis), and susceptible (S; large compatible uredinia without chlorosis or necrosis), or intermediate of any two categories (MRMS, MSS, SMS) (Roelfs, 1992; McIntosh et al., 1995; Yu et al., 2011). The weighted score of modified Cobb scale were taken as coefficient of infection as explained by Yu et al. (2011). The conversion of modified Cobb scale is explained in Supplementary Table S1.

#### Growth Chamber Assays

##### Experimental design

To confirm the disease response observed in the field, one of the resistant CRN individual (FAR14-94A-1) and one of the susceptible PRN individual (FAR14-1-1) were selected for seedling assays in the growth chamber using *Pgt* race QCCJB and HKHJC. *Pgt* isolate QCCJB is virulent on *Rpg1* but avirulent on RMRL; and HKHJC is virulent on RMRL but avirulent on *Rpg1*. Any NIL resistant to both isolates suggests the presence of a functional *Rpg1* and RMRL (*rpg4/Rpg5* genes). The parental lines, Q21861, HQ1, Pinnacle, Conlon and the susceptible controls, Harrington and Steptoe were planted for each disease reaction assay. Each entry was planted in seven cones and randomly distributed in a 12 × 7 rack.

Seedling assay was also conducted on F_2_ progeny lines and the PRN/Q21861 F_4:5_ RILs using *Pgt* race QCCJB and the further advanced F_5:6_ RIL population was screened using the two *Pgt* races QCCJB (avirulent on RMRL and virulent on *Rpg1*) and HKHJC (virulent on RMRL and avirulent on *Rpg1*). For the assay using F_2_ progenies, 94 F_2_ progenies were randomly selected and grown in 6-inch plastic cones with one seed per cone along with the PRN and Q21861 parents. The phenotypic segregation for *Pgt* race QCCJB in the F_2_ population segregated in a 3:1 ratio (resistance to susceptibility) showing a qualitative/single gene segregation. To map the single gene qualitative trait in the RIL population, two independent experimental replications of the PRN/Q21861 F_4:5_ RILs were inoculated with *Pgt* race QCCJB or HKHJC in growth chamber assay. For each independent experiment, two replication of each RIL in separate cones containing two individual seedlings were planted and arranged in a randomized complete block design. For each rep of the 126 RIL population the parental lines Q21861 and PRN, as well as Pinnacle, CRN, Conlon, Morex, Steptoe and Harrington were assayed as controls. Morex is a six-row malting barley cultivar release by the University of Minnesota that carries *Rpg1* (Kleinhofs et al., 1993). Harrington and Steptoe are wheat stem rust susceptible lines which do not harbor any known stem rust resistance genes (Brueggeman et al., 2008). For the assay using HKHJC, results were only available from a single replication of the first experiment and both replications of the second experiment.

##### Stem rust inoculation

After planting, the rack containing cones with seeds were transferred to a growth chamber (Model 7301-75-2; Caron, Marietta, OH, United States) set at a 16/8 h light/dark cycle and a day/night temperature of 21/18°C. When the primary leaves were fully expanded and the secondary leaf was still at the whorl stage (7–8 days post sowing), the seedlings were inoculated with *Pgt* race QCCJB or HKHJC using the protocol previously described by Steffenson et al. (2009) and moved to a humidity chamber. After 18 h in a dark humidity chamber at 100% relative humidity, the inoculated plants were moved back to the growth chamber set at the previously described growing condition for 14 days. At 14 days post inoculation (DPI), the infection types (ITs) were assessed using a modified 0–4 rating scale established for barley (Stakman et al., 1962; Steffenson et al., 2009). The scores were converted to a quantitative score using a conversion scale devised by Zhou et al. (2014). The conversion scale is explained in Supplementary Table S2.

### Statistical Analysis

To classify the individual as resistant or susceptible, the quantitative scores of ITs were obtained using the conversion scale of Zhou et al. (2014). Any individual with a score greater than 3 was classified as susceptible. The Pearson’s chi-square (χ^2^) goodness of fit test statistic was used to evaluate the independent segregation of resistance to susceptibility in the F_2_ and F_4:5_ RIL populations, for single or two gene segregation. The test result in the F_2_ population was used to determine the nature of inheritance of the gene/s. Since only *Pgt* race QCCJB was used to assay the F_2_ population, the inheritance of *Rrr1* was determined as a single gene required for RMRL-mediated resistance, but the inheritance of *Rrr2* was not determined. The *p*-value associated with Pearson’s χ^2^ statistics was obtained using the CHISQ.TEST function in Microsoft Excel.

### gDNA Extraction for PCR-GBS

Polymerase chain reaction-genotyping by sequencing (PCR-GBS) was used to genotype the PRN/Q12861 F_4:5_ RIL population and the parental lines PRN and Q21861, as well as Pinnacle. The gDNA used for PCR-GBS was isolated using a modified version of the 96-well plate extraction methods described by Ivanova et al. (2008) (protocol obtained from Dr. Xuehui Li’s lab, Department of Plant Sciences, NDSU). An ∼3 cm piece leaf tissue from each individual were collected in 96-well 2.2 ml deep well plates (VWR, PA, United States) and lyophilized for 24 h at -40°C. Two 4 mm stainless steel grinding balls (VWR, PA, United States) were added to each well and the samples were powdered using a Mixer Miller Type 301 tissue grinder (Retsch Gmbh & Co. KG, Germany) set at a frequency of 20/s for 3–6 min. The powdered tissue samples were homogenized in 500 μl extraction buffer (100 mM Tris base pH 8.0, 50 mM EDTA pH 8.0, 500 mM NaCl, 1.25% SDS) and incubated at 65°C for 30 min with brief vortexing every 10 min. The plates were incubated at -20°C for 10 min, then 166 μl of precipitation solution (5M ammonium acetate) was added to each sample. The samples were briefly vortexed and transferred back to -20°C for 10 min. The plates were centrifuged at room temperature at 4,000 RPM (Revolution Per Minute) for 25 min. About 400 μl of supernatant was transferred to a fresh 96-well 2.2 ml deep well plate containing 600 μl of DNA binding solution (6M Guanidine-HCl, 63% alcohol) and mixed by pipetting 3–5 times. The mixed samples were transferred to a AcroPrep^TM^ DNA filter plate stack (part# 8132, Pall Corporation, Port Washington, NY) set over 96-well 2.2 ml deep well plates and centrifuged (3,000 RPM) for 10 min at room temperature. The eluents were discarded after centrifugation followed by two rounds of filter wash using 800 μl of wash solution (10 mM Tris pH 8.0, 1 mM EDTA pH 8.0, 50 mM NaCl, 67% ethanol). The remnant wash solution after the final wash was removed by centrifugation for 10 min at 3,000 RPM. The glass plates were placed over a 1.2 ml deep well plate and 200 μl of elution buffer (10 mM Tris pH 8.0, 20 μg/ml RNase) was added to each well. Finally, 200 μl of eluted DNA was collected in 1.2 ml deep well plate by centrifugation at 3,000 RPM for 5 min. About 10–15 samples were randomly selected to check for concentration using the Qubit^®^ HS DNA kit in the Qubit^®^ 2.0 fluorometer (Invitrogen, Carlsbad, CA, United States).

### PCR-GBS Library Preparation and Ion Torrent Sequencing

A PCR-GBS SNP marker panel containing 365 barley SNP markers designed by Tamang (2017) was used in this study. The marker panels were divided into six pools as described by Tamang (2017). Six different PCR amplification reactions per sample, each representing one primer pool, were run for library preparation. Each reaction volume of 5 μl contained 1.5 μl of gDNA (DNA concentration ranging for 10 –50 ng/μl), 1 μl of 500 nM primer pool and 2.5 μl of 2× Platinum^®^ Multiplex PCR Master Mix (Life Technologies, CA, United States). The primary PCR amplifications were done in a Mastercycler Pro programmable thermocycler (Eppendorf, Hauppauge, NY, United States) with conditions set at: initial denaturation of 94°C for 10 min; followed by a touchdown step of 10 cycles of denaturation at 94°C for 20 s and annealing at 62°C for 1 min, where the temperature was decreased by 0.8°C each cycle; followed by 20 cycles of denaturation at 94°C for 20 s, annealing at 57°C for 1 min, and extension at 72°C for 1 min; ending with a final template extension at 72°C for 3 min. The PCR reactions were diluted to a volume of 20 μl using nuclease-free H_2_O. After brief vortexing and centrifugation, 5 μl of diluted PCR product from each well was aliquoted to a new 96-well plate; each well represented a single sample with a total volume of 30 μl per sample (5 μl × 6 primer pools). A barcoding PCR mix of 20 μl volume was prepared using 2 μl of pooled single genotype amplicons, 1 μl of 5 μM unique barcode primers, 150 μM of dNTPs, 250 nM of universal reverse primer, 1× GoTaq^®^ Buffer (Promega, United States) and 1 U of GoTaq^®^ DNA polymerase. The PCR amplification parameters were the same as the primary PCR. After completion of secondary PCR cycles, 15 μl of nuclease-free water was added to each well followed by a quick vortex and spin. From each well, 5 μl of samples were aliquoted and combined in a clean 1.5 ml tube (total volume = 5 μl × total number of samples) and purified using a E.Z.N.A Cycle Pure Kit (Omega Bio-tek, Inc., GA, United States). An aliquot of 2 μl from the purified pooled PCR amplicons was used for PCR-GBS library amplification in a 30 μl reaction containing 1× GoTaq^®^ Buffer, 333 μM ABC1 and P1 primer, 166 μM dNTPs and 1 U GoTaq^®^ DNA polymerase. A negative control reaction was set up without GoTaq^®^ DNA polymerase. Both reactions were run in a PCR thermocycler with parameters set at: initial denaturation of 95°C for 5 min; followed by eight cycles of denaturation at 95°C for 30 s, annealing at 62°C for 30 s, and extension at 72°C for 30 s; ending with final template extension at 72°C for 7 min. After PCR, the concentration of the Taq and No-Taq reactions were measured using the Qubit HS DNA kit with the Qubit 2.0 fluorometer. About 2 μl of reaction were run on a 1% agarose gel pre-stained with Gelred (Biotium, Hayward, CA, United States) to visualize the amplicons. No spurious band (<100 bp) were observed in the reaction with Taq and thus were processed for enrichment using Ion PI^TM^ Hi-Q^TM^ OT2 200 Kit (Life Technologies) without any further purification. The enrichment was done with the Ion OneTouch^TM^ 2 System and finally, the samples were sequenced on the Ion Torrent Personal Genome Machine (PGM) using an Ion 318^TM^ Chip following the manufacturers standard protocol.

### SNP Calling and Genotyping

The sequencing reads generated with the Ion Torrent PGM were uploaded to CLC genomics workbench v8.0 software (CLC bio, Aarhus, Denmark) to perform the quality trimming (using default parameters) and end reads trimming. The 5’ and 3’ end of each sequence was trimmed by 22 nucleotides to remove the PCR adapters (Richards et al., 2016). The trimmed reads were used for alignment using the Burrows–Wheeler Aligner maximal exact match (BWA-MEM) algorithm (Li, 2013). The sequence for each marker was obtained from the barley T3 database^1^ and used to design a fasta reference file for mapping. The SNP calling was done on the aligned BAM (Binary Alignment Map) file using the Genome Analysis Toolkit (GATK) Unified Genotyper tool with default setting for multi-sample SNP calling (Van der Auwera et al., 2013). VCFtools was used to remove individual calls with read depth of less than 6 and genotype quality less than 10 (Danecek et al., 2011). Only the SNPs reported in the iSelect 9K SNP array for the selected markers were used for mapping.

An in-house visual basic script was utilized to calculate the frequency of the reference and alternate SNP allele called for each marker in each sample. The genotype of a sample for a given marker called as homozygous for reference allele or alternate allele, if the allele frequency of either of the alleles is greater than 70%. Sample with allele frequency within 30–70% for either of the allele for a given SNP were scored as heterozygous.

### Genetic Mapping

The iSelect consensus genetic map developed by Muñoz-Amatriaín et al. (2014) was used as a reference to assign the markers to their respective barley chromosome and chromosomal position. The genotypic and phenotypic data were used to manually construct a.qdf file containing a standard format for importing marker, map and trait data into the publicly available QTL mapping software QGene v.4.3.10 (Joehanes and Nelson, 2008). The imported data were then analyzed by a composite interval mapping (CIM, Jansen and Stam, 1994; Zeng, 1994) in QGene v.4.3.10. To control the background variation, a forward cofactor selection method was used to select marker as cofactors with options, “Maximum number of cofactor” and “F to add” set at auto. Permutations of 1,000 were used to obtain a LOD threshold for an experiment-wide significance level of 0.01.

### Genetic Marker Saturation on Chromosome 5H Using PCR GBS

To saturate the region delimiting *Rrr1* a total of 24 polymorphic (Q21861 and Pinnacle) iSelect markers located between the two chromosomes 5H markers 11_21247 and 12_30162 were selected. Genotypic data on Q21861 and Pinnacle were obtained from different genotypic experiments that are publicly available in the Barley T3 database (see footnote 1). For each marker, the forward and reverse primers were designed to amplify the region containing the diagnostic SNP (Supplementary Table S5). Adaptor sequences CS1 and CS2 were attached to the forward and reverse primers, respectively, for Ion Torrent sequencing compatibility (Richards et al., 2016). The primers were multiplexed into two pools, each containing 12 markers, as recommended by Richards et al. (2016). These new pools were used to re-genotype the PRN/Q21861 F_4:5_ RILs and parental lines. The PCR-GBS library preparation, Ion Torrent sequencing, SNP calling and genetic mapping was performed as previously described. This genotypic data from the new set of markers on barley chromosome 5H was combined with the 5H marker data from the 365 marker panel.

The phenotypic score obtained from screening the F_4:5_ RILs using *Pgt* race QCCJB and HKHJC was converted to a binary scale to match the genotypic calls [score < = 3 = A (Q21861 like allele), score > 3 = B (Pinnacle like allele)] and used as the phenotypic markers *Rrr1* and *Rrr2*, respectively. A genetic map was created using MapDisto v1.7.7.0.1.1 with default minimum LOD of 3.0, rmax of 0.3 and the Kosambi mapping function (Kosambi, 1943). In order to compute a physical distance between RMRL and the iSelect marker used in this mapping, their physical positions were obtained by conducting a blast search against IBSC (International Barley Sequencing Consortium) v1 Morex genome using Viroblast in the IBSC blast server (Deng et al., 2007; Mascher et al., 2017)^2^.

### Genetic Marker Saturation Using Barley 50K iSelect SNP Array

The barley 50K iSelect SNP array was used to genotype a total of 77 PRN/Q21861 F_5:6_ RILs and their parental lines, Q21861 and Pinnacle-NIL to saturate the genetic map of barley chromosome 5H and 7H. The genotyping was performed as described by Bayer et al. (2017). The genotypic data was filtered to obtained genetic markers with minor allele frequency (maf) >0.1 and missing data <20%. The filtered genotypic data from 50K assay was combined with genotypic data from PCR-GBS to compute genetic map distance between markers in chromosome 5H and 7H using MapDisto v1.7.7.0.1.1 with default settings of minimum LOD of 3.0, rmax of 0.3 and the Kosambi mapping function (Kosambi, 1943). The genetic map distances were utilized to select non-co-segregating markers. Finally, non-co-segregating markers belonging to barley chromosome 5H and 7H, respectively, were used to compute genetic distances in MapDisto v1.7.7.0.1.1 using the aforementioned parameters. The genetic distances between selected markers were used to draw a linkage map for 5H and 7H using the publicly available software MapChart v2.32 (Voorrips, 2002).

### Candidate Genes Identification

The publicly available high-confidence genes that were annotated for IBSC RefSeq v1.0 of barley cv. Morex genome^3^ were used to identify the candidate *Rrr1* and *Rrr2* genes within the delimited region. The physical position of the genetic markers delimiting the *Rrr1* and *Rrr2* genes were used to define the physical contigs containing the candidate genes.

## Results

### Phenotypic Evaluation of NILs

In the Njoro, Kenya *Pgt* race TTKSK inoculated field nursery, the RMRL donor parent Q21861 for the PRN, showed consistent resistance responses with a median IR of trace (T) for disease severity and moderately resistant for IT (TMR; Supplementary Table S1). Disease rating data for the HQ1 NIL, the donor of the Q21861 RMRL for the CRN, was not produced at this site year, however, the previous site year screening of HQ1 in the same nursery showed a median IR of 5RMR. The IR for Pinnacle were clearly susceptible ranging from 15 to 30MSS with a median response of 25MSS. Likewise, Conlon gave a clear susceptible reaction with an IR ranging from 5MS to 25MSS and a median score of 20MS. Interestingly, a segregating disease response was observed for the 12 CRN individuals tested. However, three of the CRN individuals (FAR14-94A-1, FAR14-94A-2, FAR14-95A-2) were clearly resistant compared to the susceptible recurrent parent Conlon. FAR14-94A-1 gave a consistent disease response of 5MSMR across both replication, while FAR14-94A-2 had data from replication 2 only with a score of 5MR and FAR14-95A-2 gave a consistent disease score of 5MS across all replications (Supplementary Table S1). However, all four individuals of PRN (FAR14-1-1, FAR14-1-2, FAR14-1-3 and FAR14-1-4) were susceptible with scores comparable to its susceptible parent Pinnacle (Supplementary Table S1). The highest disease score of 30MSS was observed in FAR14-1-2 and the lowest score of 10MSS was observed in FAR14-1-4.

Inoculation of resistance parental lines Q21861 and HQ1 with *Pgt* race QCCJB exhibited low ITs (Supplementary Table S2). The susceptible parental lines, Pinnacle and Conlon were both susceptible to QCCJB with a median score of 3-2 and 3,3-, respectively. CRN (FAR14-94A-1) inoculated with QCCJB exhibited a resistance response with a median IR of 1; while the PRN (FAR14-1-1) was as susceptible as Pinnacle wild type with a median score of 3,3- (Supplementary Table S2).

The RMRL donor of the PRN, Q21861 was also highly resistant to HKHJC as it contains the *Rpg1* gene, exhibiting a median score of 0;1 (Supplementary Table S2). Likewise, the RMRL donor of the CRN, HQ1, exhibited a high median IT of 3-2 as it was selected to lack *Rpg1*. Pinnacle and Conlon were resistant to HKHJC as they are known to contain *Rpg1*. The CRN (FAR14-94A-1) as expected exhibited resistant ITs, median ITs of 1;, similar to line Q21861. However, the PRN (FAR14-1-1) was different from both of its parents, exhibiting a highly susceptible median IT of 3-3.

**FIGURE 1:**
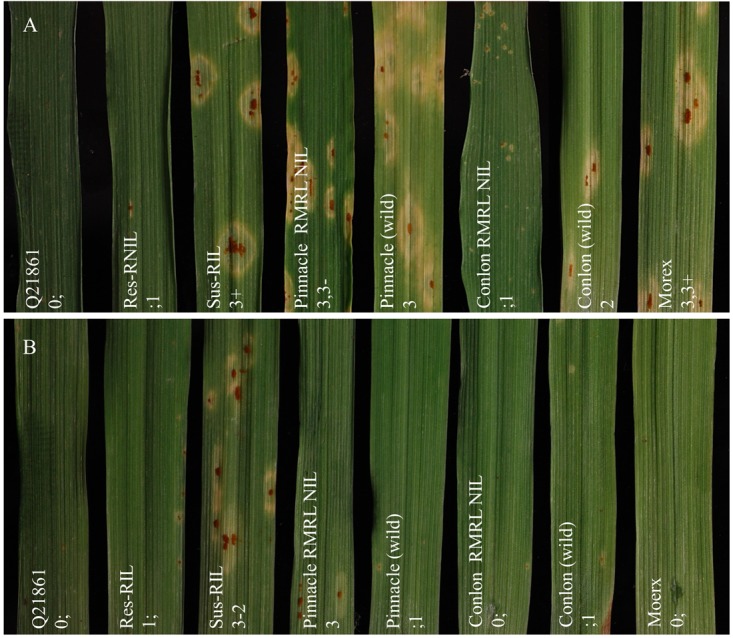
Seedling assay using *Puccinia graminis* f. sp. *tritici* (*Pgt*) races QCCJB and HKHJC. **(A)** *Pgt* race QCCJB is avirulent on barley lines containing RMRL and **(B)** *Pgt* race HKHJC is avirulent on lines containing *Rpg1*. From left: Q21861 (RMRL+, *Rpg1*+), Res-RIL [Resistant F_4:5_ Pinnacle RMRL-NIL/Q21861 RIL (RMRL+, *Rpg1*+)], Sus-RIL [Susceptible F_4:5_ Pinnacle RMRL-NIL/Q21861 RIL (RMRL+, *Rpg1*+)], Pinnacle RMRL-NIL (RMRL+, *Rpg1*+), Pinnacle wild type (RMRL-, *Rpg1*+), Conlon RMRL-NIL (RMRL+, *Rpg1*+), Conlon wild type (RMRL-, *Rpg1*+), Morex (RMRL-, *Rpg1*+). A 0–4 modified scale for barley was used to score the disease response (Stakman et al., 1962; Steffenson et al., 2009).

Ninety-four of the PRN/Q21861 F_2_ individuals were inoculated with the RMRL specific *Pgt* race QCCJB. In this assay, consistent with previous assays of the parental lines, PRN and Q21861 exhibited median ITs of 3-2 and 0;1, respectively (Supplementary Table S3). Among the F_2_ progeny, the lowest ITs were 0; and the highest were 3+ (Supplementary Table S3). Likewise, the lowest observed IR in the F_4:5_ RILs inoculated with QCCJB was 0; and the highest IR was 3, 3+ (Supplementary Table S4 and Figure 1A). The screening of F_5:6_ RILs with race HKHJC resulted in the lowest IR of 0; and the highest IR of 3+ (Supplementary Table S4 and Figure 1B).

**FIGURE 2:**
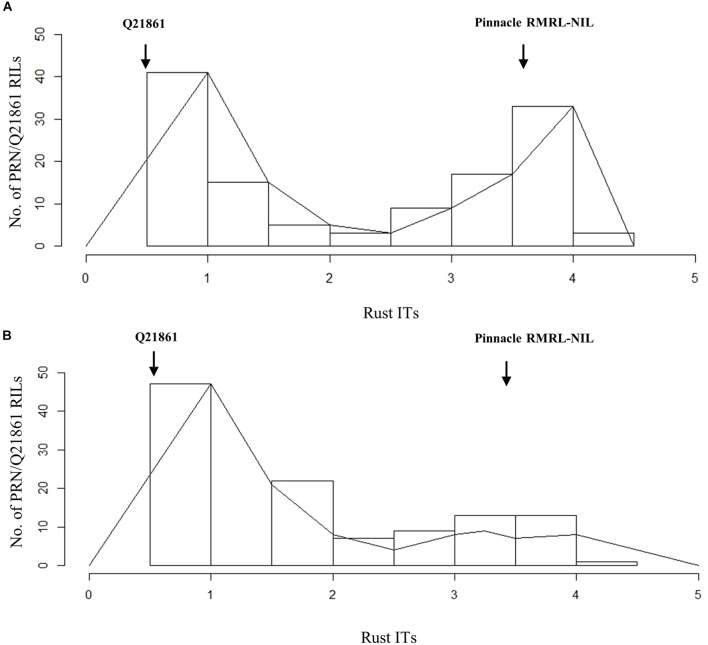
Histogram of stem rust infection types (ITs) exhibited by PRN/Q21861 RIL mapping population upon inoculation with *Pgt* races **(A)** QCCJB and **(B)** HKHJC. The rust ITs are shown on *y*-axis representing the quantitative scores obtained when using the conversion scale provided by Zhou et al. (2014).

The Pearson’s chi-square (χ^2^) goodness of fit test confirmed that the F_2_ progeny derived from the PRN/Q21861 cross, which was fixed for the Q21861 RMRL, segregated for 3 resistant: 1 susceptible, when inoculated with *Pgt* race QCCJB, suggesting monogenic inheritance of a single dominant gene required for RMRL function (Table 1). This result was further validated using the F_4:5_ RILs inoculated with QCCJB that did not significantly deviate from 1 resistant: 1 resistant to susceptible ratio (Figure 2) as expected for a single gene segregation in the RIL population [chi-square (χ^2^) *p*-value >0.05, Table 1]. The segregation ratio of IRs of the F_5:6_ RIL population inoculated with HKHJC did not comply with 1:1 (resistance:susceptibility) but fit a 3:1 ratio (resistance:susceptibility) (Table 1). The 3:1 segregation ratio for resistance to susceptibility in the RIL population inoculated with *Pgt* race HKHJC suggested that two genes with a complementary gene function are required for functional *Rpg1*-mediated resistance in the presence of a fixed RMRL.

**Table 1:** Chi-square goodness-of-fit test to assess the segregation for resistance to susceptibility in different generation of progenies generated from Pinnacle RMRL-NIL/Q21861.

*Pgt* race	Generation^a^	Experiment	Rep	No. of individuals^b^		Expected segregation ratio^c^	*p*-value^d^
				Resistant	Susceptible		
QCCJB	F_2_	1	1	63	31	3:1	0.07
	F_4:5_	1	1	81	38	1:1	8E-5
			2	75	44		0.004
		2	1	50	61		0.29
			2	54	53		0.92
HKHJC	F_5:6_	1	1	74	38	3:1	0.03
		2	1	79	30		0.54
			2	83	27		0.91

^a^*The generation of progenies derived from Pinnacle RMRL-NIL/Q21861 cross that was screened with given *Pgt* race. ^*b*^The number of individuals that gave resistance or susceptible response when inoculated with given *Pgt* race. ^*c*^The expected Mendelian segregation ratio for resistance to susceptible in given assay. A trait governed by a single trait will segregate for 3:1 and 1:1 ratio in F_*2*_ and RIL, respectively. In a RIL, two genes that are complementary for a single phenotype will give a phenotypic segregation 3:1 for resistance to susceptibility. ^*d*^*p*-Value >0.05 represents that dataset that fit the expected Mendelian segregation ratio for resistance to susceptibility*.

### Genetic Mapping of Rrr1 and Rrr2

**FIGURE 3:**
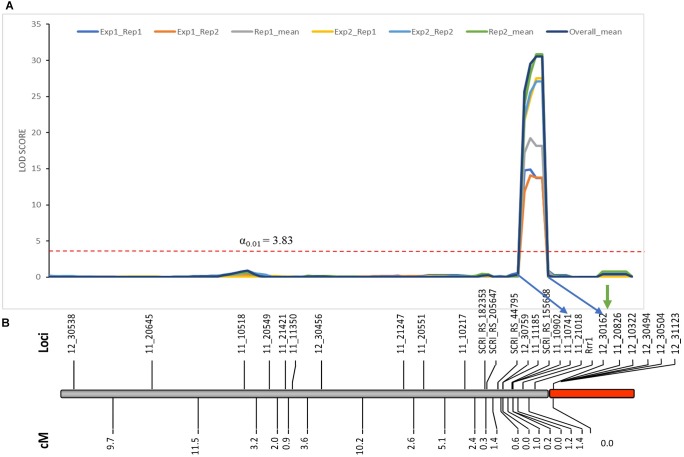
**(A)** Genetic mapping of *Rrr1* using the composite interval mapping in Qgene v4.3.0. on barley chromosome 5H. The dotted line represents a LOD threshold obtained at experiment-wide significance level of 0.01 after 1,000 permutations test. **(B)** The genetic map of barley chromosome 5H developed using the polymorphic markers available in our dataset. A default minimum LOD score of 3.0, rmax of 0.3 and Kosambi mapping function was used in MapDisto v1.7.7.0.1.1 to compute genetic map distance (cM) based on recombination frequency of 126 Q21861/Pinnacle-RMRL-NIL derived RILs. The PCR-GBS 365 marker panel contained no SNP markers distal to *Rrr1* that were polymorphic between Q21861 and Pinnacle RMRL-NIL (represented by red color). The green arrow represents the position of RMRL [obtained using physical position (Table 2)].

**FIGURE 4:**
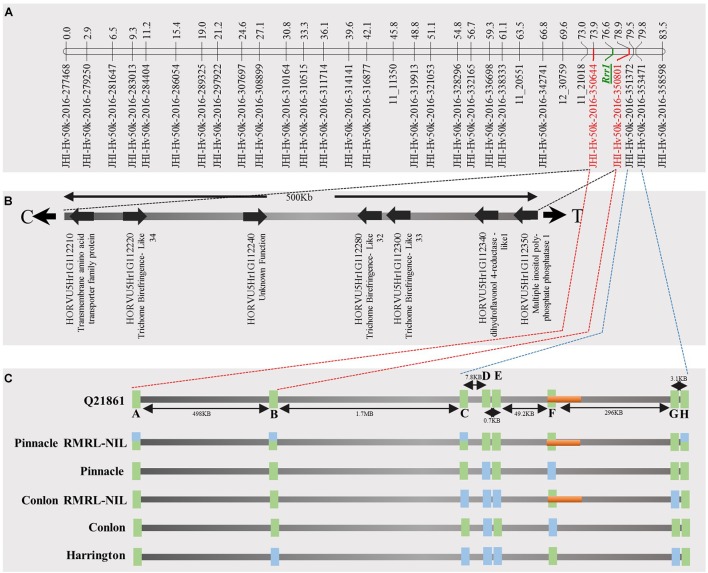
Genetic marker saturation of barley chromosome 5H using barley 50K iSelect SNP array markers and identification of candidate genes for *Rrr1*. **(A)** The recombination frequency of 77 Q21861/Pinnacle RMRL-NIL derived RILs was used to compute genetic map distance between 84 non-co-segregating genetic markers and *Rrr1* phenotypic markers using a Kosambi mapping function in MapDisto v.1.7.7.7. The genetic map distance was used to draw a linkage map in software MapChart v2.32. *Rrr1* was delimited to a genetic distance of ∼5.01 cM between flanking markers JHI-Hv50K-2016-350644 and JHI-Hv50K-2016-350801. **(B)** The physical position of the genetic markers flanking *Rrr1* was used to identify seven high-confidence annotate genes from IBSC RefSeq v1.0 of barley cv. Morex genome (http://webblast.ipk-gatersleben.de/barley_ibsc/downloads/). C and T at the end of the horizontal line indicate the direction of centromere and telomere, respectively. **(C)** Allele analysis of marker flanking *Rrr1* (represented by red dotted lines) and RMRL (represented by blue dotted lines). 50K iSelect SNP markers are represented as A (JHI-Hv-50K-2016-350644), B (JHI-Hv-50K-2016-350801), C (JHI-Hv-50K-2016-351372), D (JHI-Hv-50K-2016-351438), E (JHI-Hv-50K-2016-351449), F (JHI-Hv-50K-2016-351467), G (JHI-Hv-50K-2016-351585) and H (JHI-Hv-50K-2016-351627). The green bars represent a SNP marker with a Q21861 allele and blue color represents the alternate allele, while semi-blue-green color represents a heterozygous call for a given marker. The orange bar represents a physical region containing RMRL.

As expected from the Pearson’s chi-square (χ^2^) goodness of fit test, a single gene required for *rpg4*-mediated resistance, designated *Rrr1*, was mapped to the telomeric region of barley chromosome 5H, ∼5 cM proximal to RMRL (Figure 3 and Table 2). The genotypic data obtained from the 365 PCR-GBS SNP marker panel was utilized to map the *Rrr1* gene to a region of barley chromosome 5H, flanked by the SNP markers 11_20551 (130.93 cM iSelect consensus map position) and 12_30162 (156.7 cM iSelect) based on the consensus map generated by Muñoz-Amatriaín et al. (2014). After saturating the delimited *Rrr1* region using a custom PCR-GBS SNP panel designed from SNPs mined from the recently released barley 50K iSelect markers, the *Rrr1* region was further delimited to an ∼1.4 cM region distal to iSelect marker 11_21018 (153.74 cM iSelect position) (Figures 3, 4). Even though all the markers used to construct the linkage map were from the chromosome 5H, MapDisto generated two different chromosome 5H linkage groups due to the fixed Q21861 RMRL introgression in the PRN, which was monomorphic distal of *Rrr1*.

**Table 2:** Genetic and physical location of markers associated with *Rrr1* and *Rrr2*.

iSelect marker	Chr^a^	cM^b^	cM^c^	Bp Start^d^	Bp End^d^
11_21018	5H	153.74	N/A	636679883	636679883
JHI-Hv50k-2016-350644		N/A	N/A	638453433	638453433
12_30162		156.7	N/A	638951179	638951179
JHI-Hv50k-2016-350801		N/A	N/A	638951253	638951253
JHI-Hv50k-2016-351467		N/A	N/A	640766892	640766892
RMRL^e^		N/A	N/A	640765916	640837301
11_20826	5H	161.41	N/A	642764921	642764921
11_21419	7H	0	N/A	737146	737146
*Rpg1*^f^		N/A	N/A	3257770	3451493
JHI-Hv50k-2016-441751		N/A	N/A	5918682	5918682
JHI-Hv50k-2016-442218		N/A	N/A	6623223	6623223
12_30880		63.28	54.39	67729137	67729137

^a^*Chromosome assignment based on iSelect consensus map (Muñoz-Amatriaín et al., 2014) and POPSEQ map (Mascher et al., 2013) and the IBSC v2 barley genome sequence (Mascher et al., 2017). ^*b*^Chromosomal position based on iSelect consensus map developed by Muñoz-Amatriaín et al. (2014). ^*c*^Chromosomal position based on POPSEQ map developed by Mascher et al. (2013). ^*d*^Physical position in IBSC v1 genome sequence of barley cultivar Morex (Mascher et al., 2017). ^*e*^GenBank accession # EU878778 used as a query for RMRL (Brueggeman et al., 2008). ^*f*^GenBank accession # AF509750 used as a query for *Rpg1* (Brueggeman et al., 2002)*.

**FIGURE 5:**
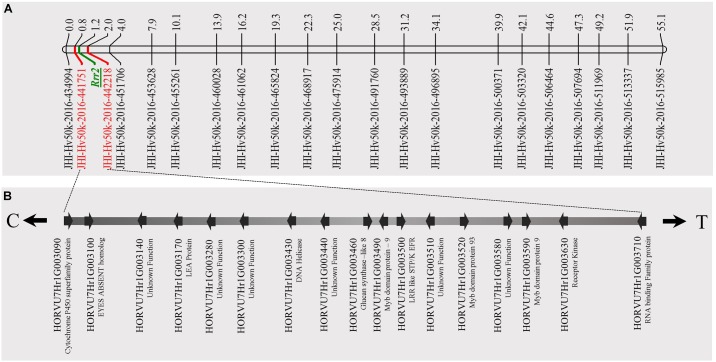
Genetic map of barley chromosome 7H using the barley 50K iSelect SNP array markers and identification of candidate genes for *Rrr2*. **(A)** The genetic map distance of 71 non-co-segregating markers in barley chromosome 7H was computed based on the recombination frequency of 77 Q21861/Pinnacle RMRL-NIL derived using a Kosambi mapping function in MapDisto v.1.7.7.7. The publicly available software MapChart v2.32 was used to draw a linkage map using the obtained genetic map distances. *Rrr2* was delimited to a genetic distance of ∼1.8 cM between the flanking markers JHI-Hv50K-2016-441751 and JHI-Hv50K-2016-442218. **(B)** The physical position of the genetic markers flanking *Rrr2* were used to identify seventeen high-confidence annotate genes from the IBSC RefSeq v1.0 of barley cv. Morex genome (http://webblast.ipk-gatersleben.de/barley_ibsc/downloads/). C and T at the end of the horizontal line indicate the direction of centromere and telomere, respectively.

The genetic mapping of the genes segregating for *Pgt* race HKHJC resistance in the PRN/Q21861 RIL population as suspected by the 3:1 segregation ratio identified two genes/loci with duplicate dominant epistasis required for *Rpg1*-mediated resistance only in the presence of a fixed RMRL. One of the loci mapped co localized with *Rrr1*, while the other locus, the *Rrr2* gene, mapped to the telomeric region of barley chromosome 7H (Figure 5), flanked by markers 11_21419 (0 cM iSelect position) and 12_30880 (63.28 cM iSelect position). This result confirmed that *Rrr1* is required for RMRL function against *Pgt* races QCCJB and TTKSK mediated resistance. However, surprisingly *Rrr1* is also required for *Rpg1*-mediated resistance, but only in the presence of the RMRL, but *Rrr2* can confer *Rpg1* mediated resistance in the presence or abasence of *Rrr1* because of the duplicate gene action.

### Genetic Marker Saturation of *Rrr1* and *Rrr2* Using Barley iSelect 50K

A total of 85 and 71 non-co-segregating markers were identified from the Barley iSelect 50K data and utilized to saturate of barley chromosomes 5H *Rrr1* and 7H *Rrr2* loci, respectively. After marker saturation *Rrr1* was delimited to ∼5.01 cM based on recombination frequency of 77 PRN/Q21861 derived RILs between the flanking markers JHI-Hv50k-2016-350644 and JHI-Hv50k-2016-350801. The markers flanking *Rrr1* delimited an ∼0.5 MB physical region, slightly proximal (∼1.8 MB) to RMRL on barley chromosome 5H (Table 2 and Figure 4A). Likewise, *Rrr2* was delimited to an ∼1.27 cM genetic region flanked by the markers JHI-Hv50k-2016-441751and JHI-Hv50k-2016-442218 corresponding to a physical region of ∼0.7 MB, slightly distal (∼3.1 MB) to *Rpg1* (Table 2 and Figure 5A). Based on the physical region delimiting *Rrr1* and *Rrr2*, 7 high-confidence genes were identified as candidate for *Rrr1* (Figure 4B and Supplementary Table S6); while 17 high-confidence genes were identified as *Rrr2* candidates (Figure 5B and Supplementary Table S6).

## Discussion

The emergence of the highly virulent *Pgt* race TTKSK and subsequent wheat and barley yield losses in two continents due to epidemics caused by this race or its lineage leaves production vulnerable if or when it is introduced to other regions (Singh et al., 2011; Steffenson et al., 2017). The only effective *Pgt* race TTKSK resistance in barley is the RMRL. The RMRL contains three tightly linked genes, *Rpg5*, *HvRga1* and *HvAdf3*, which are inherited as a single genetic unit and function together for resistance (Brueggeman et al., 2008; Steffenson et al., 2009). *Rpg5*-specific markers have been used to efficiently introgress RMRL into elite barley (Derevnina et al., 2014) in several breeding programs, yet, it has been found to be ineffective when interacting with genes in some genetic backgrounds. We used *Rpg5* allele specific molecular markers to incorporate the Q21861 RMRL into Upper Midwestern US malting varieties, developing NILs of the varieties Conlon and Pinnacle. Saturation of the NILs with SNP markers (Table 2 and Figure 4) defined the Q21861 introgression in the PRN and CRN validating successful RMRL introgression. However, only the CRN exhibited resistance to the RMRL specific *Pgt* races QCCJB and TTKSK comparable to Q21861. The PRN remained susceptible, suggesting the requirement of additional gene/s for resistance that are functionally polymorphic between the resistant barley accession Q21861 and the susceptible barley variety Pinnacle.

**FIGURE 6:**
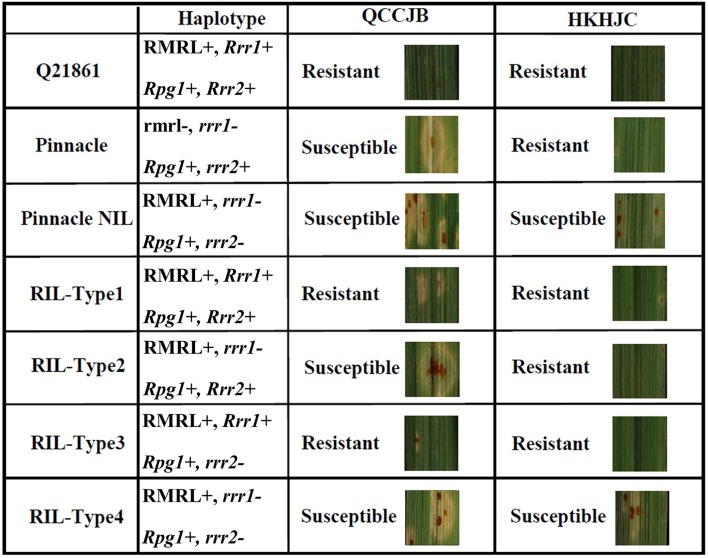
Seedling assay using *Pgt* race QCCJB and HKHJC showing disease response on barley containing different combination of RMRL, *Rpg1*, *Rrr1* and *Rrr2* alleles. QCCJB is virulent on *Rpg1* and HKHJC is virulent on RMRL. Pinnacle is the recurrent parent and Q21861 is the RMRL donor of the Pinnacle RMRL-NIL. Four different allelic combinations of the two additional genes required for stem rust resistance, *Rrr1* and *Rrr2*, occur in the Pinnacle RMRL-NIL/Q21861 progeny. *Rrr1* is required to confer RMRL mediated QCCJB resistance (Q21861, RIL-Type1 and RIL-Type2). *Rpg1* mediated resistance is independent of *Rrr1* and *Rrr2* in absence of RMRL (Pinnacle). The introgression of RMRL into the Pinnacle background necessitates the presence of either a functional *Rrr1* or *Rrr2* to confer *Rpg1* mediated HKHJC resistance (Q21861, RIL-Type1, RIL-Type2, RIL-Type3). *Rpg1* mediated HKHJC resistance in RIL-Type2 and RIL-Type3 shows that *Rrr1* and *Rrr2* have a complementary gene function in the *Rpg1*-mediated resistance pathway.

To identify the additional gene required for RMRL resistance, designated *Rrr1* (required for *rpg4*-mediated resistance 1), present in barley line Q21861, the PRN was crossed with Q21861 to develop a RIL mapping population that was fixed for RMRL and the *Rpg1* stem rust resistance gene. Since, Q21861 and the PRN share identical *Rpg1* and *Rpg5* alleles, the loci segregating for resistance/susceptibility in the RIL mapping population would be associated with any additional gene/s required for RMRL stem rust resistance. The responses to *Pgt* race QCCJB on the PRN/Q21861 RIL population segregated as a single dominant gene and mapped to the sub telomeric region of the long arm of barley chromosome 5H, ∼5 cM proximal to RMRL (Figures 3, 4). Because *Rpg1* and *rpg4* are distinct genes that independently assort on separate chromosomes, it was unexpected when we observed that the RMRL introgression into the Pinnacle background resulted in the suppression of *Rpg1*-mediated resistance against *Pgt* race HKHJC. However, because both *Rpg1* and RMRL function together in the Q21861 background, we hypothesized that additional loci/genes from Q21861 should also segregate that allow for both resistances to function harmoniously in the same genetic background. The responses of *Pgt* race HKHJB on the RIL population segregated as two dominant genes (3 resistant: 1 susceptible) and one mapped to the *Rrr1* region and the other mapped to the telomeric region of the short arm of barley chromosome 7H distal of *Rpg1* and was designated *Rrr2*. These data determined that *Rrr1* is required for RMRL function but is also required for *Rpg1*-mediated resistance but only in the presence of RMRL. However, the segregation ratio for resistance to susceptibility in response to HKHJB suggested that *Rrr1* and *Rrr2* complement each other for *Rpg1*-mediated resistance when RMRL is present (Figure 6 and Table 1). Thus, the PRN recovered both the non-functional allele of *Rrr1* and *Rrr2* and failed to confer RMRL- or *Rpg1*-mediated stem rust resistance. Thus, it is apparent that Pinnacle and Q21861 have functionally polymorphic *Rrr1* and *Rrr2* alleles (Figure 4).

The data shows that the PRN contains non-functional *rrr1* and *rrr2* alleles, thus Q21861 carries the dominant functional *Rrr1* and *Rrr2* alleles. Interestingly, Pinnacle confers *Rpg1*-mediated resistance despite lacking the functional *Rrr1* or *Rrr2* suggesting that *Rpg1*-mediated resistance is independent of *Rrr1* and *Rrr2*, in the absence of RMRL. The introgression of RMRL in barley genomes requires *Rrr1* to function and if RMRL is to be pyramided with *Rpg1* then *Rrr1* must be present for *Rpg1* to maintain its resistance. Although, when pyramiding these resistances to achieve the broad resistance like that found in line Q21861, which is effective against all known *Pgt* races, breeders must maintain *Rrr1* in their lines. Interestingly, the *Rrr2* gene can confer *Rpg1*-mediated resistance in the presence of RMRL, despite presence or absence of *Rrr1*, but does not complement RMRL function in the absence of *Rrr1* suggesting that these two genes do not have a complete duplicate dominant epistatic function. However, the complex suppression of *Rpg1*-mediated resistance by the presence of RMRL suggests that the two resistance mechanisms certainly have some level of interaction. This has also been exemplified by fast neutron irradiation-induced mutants that we developed with disrupted RMRL and *Rpg1*-mediated resistances (Unpublished).

Genetic and mutational analysis of diverse pathosystems including barley, tomato and Arabidopsis have characterized genes that are quintessential for the function of *R*-gene mediated defense responses (Torp and Jørgensen, 1986; Freialdenhoven et al., 1994; Aarts et al., 1998; Feys and Parker, 2000; Glazebrook, 2001). Utilizing fast neutron irradiated Arabidopsis ecotype Col-1 mutations were produced in the *non-race-specific disease resistance*, *NDR1*, and *enhanced disease susceptibility*, *EDS1* genes which showed their imperative roles in conferring disease resistance to biotrophic oomycetes and bacterial pathogens conferred by distinct classes of *R* genes (Aarts et al., 1998; Li et al., 2001). Another study using barley mutants showed a role of two genes *Rar1* and *Rar2* in executing a race specific resistance by the gene *Mla12* against the fungal pathogen *Erysiphe graminis* f. sp. *hordei* (Torp and Jørgensen, 1986; Freialdenhoven et al., 1994). A mutagenesis study in tomato identified a gene *Rcr3* that is required for *Cf-2* mediated leaf mold resistance caused by *Cladosporium fulvum* (Dixon et al., 2000). However, many of these genes are highly conserved across genotypes unlike the *Rrr1* and *Rrr2* genes, which certainly have functionally polymorphic alleles within the primary barley germplasm pool. Thus, many of these conserved genes could only be discovered by mutational studies that developed synthetic polymorphism that was not present in the primary germplasm pools.

A previous study utilized fast neutron irradiation of barley variety Morex generated the required for *P. graminis* resistance, *Rpr1* mutant that disrupted *Rpg1*-mediated resistance (Zhang et al., 2006). *Rpr1* was characterized utilizing a Q21861/*Rpr1* mapping population which mapped the gene to a region of barley chromosome 4H. Microarray analysis of the mutant and wild type identified three deleted genes in that region, and a putative serine/threonine protein kinase-like protein was considered as a strong candidate for *Rpr1* gene. Another study by Gill et al. (2016) used gamma-irradiation to develop six independent mutants in the cv Morex background designated *Rpr2-7* that also compromised *Rpg1*-mediated resistance. Utilizing a Q21861 X *rpr2* F_2_ population *Rpr2* was mapped to the centromeric region of chromosome 6H. However, these studies did not report genes on chromosome 5H and 7H that function in stem rust resistance, thus are distinct from *Rpr1* and *Rpr2*. Also, these studies utilized irradiation to produce artificial functional polymorphism suggesting that these genes are probably conserved resistance components. However, *Rrr1* and *Rrr2* are functionally polymorphic in the primary barley germplasm pool and more importantly in breeding programs; thus, our study is important to address issues that would arise when pyramiding *Rpg1* and RMRL. It will be important to identify the *Rrr1* gene and identify diagnostic markers that could be used in combination with *Rpg5* specific markers for effective marker assisted selection.

Polymerase chain reaction-GBS allowed us to utilize a larger number of RIL individuals to obtain a rough genetic map of the region containing *Rrr1* and *Rrr2*. Then, the prior knowledge of RIL genotypes based on the PCR-GBS data allowed for the efficient use of the barley 50K iSelect assay by selecting informative RIL individual to develop a genetic map with higher marker saturation. Utilizing the annotated barley genome sequence the physical region containing *Rrr1* was delimited to ∼0.5 Mb containing seven HC genes and *Rrr2* to ∼0.7 Mb containing 17 HC genes (Supplementary Table S5). To prioritize the candidates *Rrr1* and *Rrr2* genes we searched for homologous or similar function genes since our mapping results suggests a somewhat duplicate dominant epistatic action of *Rrr1* and *Rrr2* in regard to *Rpg1*-mediated resistance in the presence of RMRL. Interestingly, this study identified gene families in *Rrr1* and *Rrr2* that are not homologous but have been shown to be involved in plant immune response through cell wall modifications. Out of the seven *Rrr1* candidate genes, three belong to the Trichrome birefringence like (TBL) family that function both as positive or negative regulators of plant defense responses depending upon the pathosystem. A powdery mildew resistance gene *PMR5* (Powdery mildew resistant 5) was identified as a member of the TBL family that contributed to powdery mildew susceptibility, possibly through pectin acetylation (Vogel et al., 2004; Lim, 2013). In contrast, two TBL proteins in rice, *OsTBL1* and *OsTBL2*, contributed to resistance against leaf blight through xylan modification (Gao et al., 2017). Likewise, a Glucan synthase-like 8 (GSL-8) *Rrr2* candidate gene belongs to a GSL family that was shown to be involved in both positive and negative regulation of disease resistance in plants through cell wall modification (Nishimura et al., 2003; Ellinger and Voigt, 2014; Chowdhury et al., 2016). In Arabidopsis the powdery mildew resistance gene 4 (*pmr4*) is a mutant of the GSL-5 (*AtGsl5*) gene that was shown to enhanced resistance to the powdery mildew pathogen *Golovinomyces cichoracearum* (Nishimura et al., 2003). *PMR4* was hypothesized to induce susceptibility by providing negative feedback for SA accumulation after callose induction upon early detection of the pathogen. Contrary to *pmr4*, the *HvGsl6* (barley glucan synthase-like 6), a close homolog of *AtGsl6* was shown to reinforce resistance against barley powdery mildew *Blumeria graminis* f. sp. *hordei* through callose deposition at the site of infection (Chowdhury et al., 2016). Since the genes belonging to TBL and GSL family have been shown to be involved in regulation of the plant immune response through cell wall modification and *Rrr1* and *Rrr2* have duplicate dominant gene action in *Rpg1*-mediated stem rust resistance, it is possible that TBL is able to compensate the loss of GSL to restore *Rpg1* resistance, despite TBL and GSL having different modes of cell wall modification. Based on this hypothesis, the genes belonging to TBL and GSL could be prioritized as top candidates for *Rrr1* and *Rrr2*, respectively. However, we could not rule out the possibility of other genes as candidates for *Rrr1* and *Rrr2* especially other genes in the region with known defense function including the receptor like kinases present at the *Rrr2* locus. Thus, post-transcriptional gene silencing and characterization of other stem rust resistance mutants are being utilized to validate the *Rrr1* and *Rrr2* candidate genes.

At the functional level plant immunity responses are typically characterized into either one of two tiers of the immunity responses. The first line of defense is known as pathogen-associated molecular pattern (PAMP)-triggered immunity (PTI) and the second and higher amplitude defense is known as effector-triggered immunity (ETI) (Jones and Dangl, 2006; Thomma et al., 2011). The PTI responses are activated early at the cell surface by membrane localized pattern recognition receptors (PRRs), which recognize conserved microbial molecules like bacterial flagellin or fungal chitin. ETI responses are typically later and generally rely on nucleotide binding–leucine-rich repeat (NLR) immunity receptors that recognize pathogen proteinaceous effectors that trigger resistance signaling pathways resulting in resistance responses. The ETI responses typically induce localized programmed cell death known as the hypersensitive response (HR). However, some resistance mechanisms are difficult to characterize under these definitions and two such mechanisms are the RMRL and *Rpg1*-mediated resistance responses.

The stem rust resistance gene *Rpg1* was cloned and characterized by a positional cloning approach and shown to have an atypical R-protein structure consisting of tandem protein kinase domains (Brueggeman et al., 2002). Since the identification of *Rpg1*, it has been shown that 5 min post inoculation with avirulent *Pgt* race MCCF, the RPG1 protein is phosphorylated and degrades within 24 h and both modifications are required for resistance (Nirmala et al., 2010). The AVR-RPG1 effector proteins from the pathogen have since been identified and are present in the spore coat (Nirmala et al., 2011), suggesting that the interaction occurs prehaustoria formation. Interestingly, the RPG5 protein contains an STPK domain with homology to the RPG1 STPK gene family (Brueggeman et al., 2006) and may also elicit early defense responses similar to RPG1. However, the Rpg5 PK domain follows the “integrated decoy” or “integrated sensory domain” model (Cesari et al., 2014; Wu et al., 2015), which implies that it is possibly acted upon by an effector that is secreted by the pathogen and is possibly activated by an early recognition event as well. We hypothesize that both wheat stem rust resistance mechanisms in barley, *Rpg1*- and RMRL, rely on early prehaustorial recognition that possibly have some overlapping signaling mechanisms that when present together can inhibit each other in the absence of *Rrr1* and/or *Rrr2*.

## Author Contributions

RS and RB designed the experiments, performed the stem rust assay, and wrote the manuscript. RB and PG developed the NIL and RIL populations. RS, TG, and AA-H constructed libraries for PCR-GBS and assisted in the experiments. RS did the bioinformatic and statistical data analysis, and genetic mapping.

## Conflict of Interest Statement

The authors declare that the research was conducted in the absence of any commercial or financial relationships that could be construed as a potential conflict of interest.

## Footnotes

1 https://triticeaetoolbox.org/barley/

2 http://webblast.ipk-gatersleben.de

3 http://webblast.ipk-gatersleben.de/barley_ibsc/downloads/
